# Dynamic estimation of specific fluxes in metabolic networks using non-linear dynamic optimization

**DOI:** 10.1186/s12918-014-0132-0

**Published:** 2014-12-03

**Authors:** Dominique Vercammen, Filip Logist, Jan Van Impe

**Affiliations:** KU Leuven, BioTeC - Chemical and Biochemical Process Technology and Control & OPTEC - Center of Excellence: Optimization in Engineering, Department of Chemical Engineering, Willem de Croylaan 46/2423, Leuven, 3001 Belgium

**Keywords:** Dynamic metabolic flux analysis, B-spline parameterizations, Non-linear optimization, Parameter estimation

## Abstract

**Background:**

Metabolic network models describing the biochemical reaction network and material fluxes inside microorganisms open interesting routes for the model-based optimization of bioprocesses. Dynamic metabolic flux analysis (dMFA) has lately been studied as an extension of regular metabolic flux analysis (MFA), rendering a dynamic view of the fluxes, also in non-stationary conditions. Recent dMFA implementations suffer from some drawbacks, though. More specifically, the fluxes are not estimated as specific fluxes, which are more biologically relevant. Also, the flux profiles are not smooth, and additional constraints like, e.g., irreversibility constraints on the fluxes, cannot be taken into account. Finally, in all previous methods, a basis for the null space of the stoichiometric matrix, i.e., which set of free fluxes is used, needs to be chosen. This choice is not trivial, and has a large influence on the resulting estimates.

**Results:**

In this work, a new methodology based on a B-spline parameterization of the fluxes is presented. Because of the high degree of non-linearity due to this parameterization, an incremental knot insertion strategy has been devised, resulting in a sequence of non-linear dynamic optimization problems. These are solved using state-of-the-art dynamic optimization methods and tools, i.e., orthogonal collocation, an interior-point optimizer and automatic differentiation. Also, a procedure to choose an optimal basis for the null space of the stoichiometric matrix is described, discarding the need to make a choice beforehand. The proposed methodology is validated on two simulated case studies: (*i*) a small-scale network with 7 fluxes, to illustrate the operation of the algorithm, and (*ii*) a medium-scale network with 68 fluxes, to show the algorithm’s capabilities for a realistic network. The results show an accurate correspondence to the reference fluxes used to simulate the measurements, both in a theoretically ideal setting with no experimental noise, and in a realistic noise setting.

**Conclusions:**

Because, apart from a metabolic reaction network and the measurements, no extra input needs to be given, the resulting algorithm is a systematic, integrated and accurate methodology for dynamic metabolic flux analysis that can be run online in real-time if necessary.

**Electronic supplementary material:**

The online version of this article (doi:10.1186/s12918-014-0132-0) contains supplementary material, which is available to authorized users.

## Background

Metabolic network models describing the biochemical reaction network and material fluxes inside microorganisms open interesting routes for the model-based optimization of bioprocesses. The estimation of these fluxes is called *metabolic flux analysis (MFA)*. Based on measurements of exchange fluxes between the environment and the cell, and possibly thermodynamic, physiological, statistical [1] or loop-law constraints [2] and/or measurements from ^13^C labeling experiments, an accurate estimate of the full set of fluxes can be obtained (e.g., [3,4]). *Dynamic metabolic flux analysis (dMFA)* has lately been studied as an extension of regular MFA, rendering a dynamic view of the fluxes, also in non-stationary conditions [5]. Recent dMFA implementations suffer from some drawbacks, though. In this work, a new methodology based on a B-spline parameterization of the fluxes is presented. These are estimated using state-of-the-art dynamic optimization methods and tools, i.e., orthogonal collocation, an interior-point optimizer and automatic differentiation. The resulting algorithm is also fully contained, resolving the fact that in previous methods, a choice of the set of free fluxes was required. As will be shown, the choice of this set has a significant influence on the resulting estimates, highlighting the need for a more reasoned determination of this set. In this algorithm this set of free fluxes is chosen optimally, alleviating the need for an a priori (non-optimal) choice and improving the estimates.

In the Background section, the basics of metabolic reaction networks are covered, together with the derivation of the dMFA model structure. Existing implementations of dMFA are described, along with their features and drawbacks. The main contribution of this work is in the Methods section. Here, the proposed methodology of incremental flux estimation using B-splines is described. This methodology is validated on the case studies in the Results and discussion section. Finally, the Conclusions section summarizes the main results of this work.

### Metabolic reaction networks

**Figure 1:**
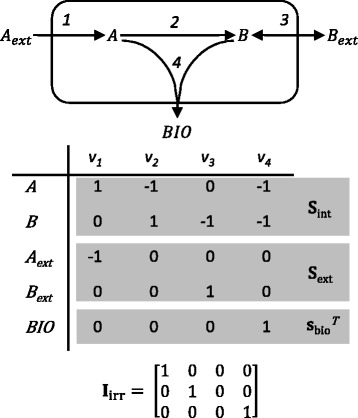
**Example of metabolic reaction network, stoichiometric matrix and irreversibility matrix.** A small-scale example of a metabolic reaction network with 4 fluxes, 2 intracellular metabolites, 2 extracellular metabolites and biomass. The number of free fluxes for this network is 2. In the middle is the corresponding stoichiometric matrix, split up into the parts for intracellular and extracellular metabolites, and biomass, and at the bottom is the irreversibility matrix for this network.

A *metabolic reaction network* represents (a subset of) all metabolic reactions which occur inside a cell [6]. In these networks, *mmetabolites*, both intracellular and extracellular, are connected to each other through *nreactions*, which can be *intracellular reactions* or reactions between the cell and the environment, so-called *exchange reactions*. The reaction rates of these reactions, the so-called *fluxes*, are summarized in the (*n*×1) flux vector **v**. The metabolites can be further subdivided into *m*_int_*intracellular metabolites* and *m*_ext_*extracellular metabolites*. Growth of the cell is usually represented as a pseudo-reaction to biomass, which is defined as an additional extracellular compound, rendering *m*_ext_+1 extracellular metabolites in total. All reactions are also classified as being reversible or irreversible, based on thermodynamic information. This results in *n*_irr_ irreversible reactions and *n*_rev_ reversible reactions. The information embedded in this network can be represented by the *stoichiometric matrix***S** of dimension (*m*+1×*n*), which contains the stoichiometric coefficients of all reactions. In particular, the element **S**_*ij*_ at row *i* and column *j* contains the stoichiometric coefficient of metabolite *i* in reaction *j*. This stoichiometric matrix can be further partitioned into **S**_int_, **S**_ext_ and ${\mathbf{s}}_{\mathrm{bio}}^T$, which are the row(s) corresponding to intracellular and extracellular metabolites, and biomass, respectively. To also describe the irreversibilities in matrix form, an (*n*_irr_×*n*) irreversibility matrix **I**_irr_ is set up, which selects the irreversible fluxes from the full set of fluxes. A small network with corresponding stoichiometric and irreversibility matrices is shown in Figure 1. For this network, *m*_int_=*m*_ext_=2 and *n*=4. The **I**_irr_ matrix is made up of three rows, as there are three irreversible fluxes. Every row contains a 1 at one of the columns corresponding to an irreversible flux.

### Modeling of intracellular dynamics

By writing the dynamic mass balances for all intracellular metabolites, the following system of ordinary differential equations (ODEs) arises:

(1) $$ \frac{d{\mathbf{c}}_{\mathrm{int}}(t)}{dt}={\mathbf{S}}_{\mathrm{int}}\cdotp \mathbf{v}(t)-\mu (t)\cdotp {\mathbf{c}}_{\mathrm{int}}(t) $$

with *t* the time [h], **c**_int_ the (*m*_int_×1) vector of intracellular concentrations [mmol/gDW], **S**_int_ the *m*_int_ rows of the stoichiometric matrix which correspond to the intracellular metabolites, **v** the (*n*×1) vector of specific fluxes [mmol/gDW/h] and *μ* the scalar specific growth rate of the organism [1/h] which equals the flux of the pseudo-reaction to biomass. The first term on the right hand side is the *reaction term*, the second term is a *dilution term* which arises due to the growth of the biomass. To get a fully defined model, expressions for all intracellular fluxes are needed.

### Multi-scale model for microbial dynamics

The intracellular model (Equation (1)) can be combined with a description for the extracellular dynamics, rendering a *multi-scale model* describing both intracellular and extracellular concentration variables:

(2) $$ \begin{array}{lcrr}\frac{d{\mathbf{c}}_{\mathrm{ext}}(t)}{dt}& =& {\mathbf{S}}_{\mathrm{ext}}\cdotp \mathbf{v}(t)\cdotp {c}_{\mathrm{bio}}(t)& \end{array} $$

(3) $$ \begin{array}{lcrr}\frac{dc_{\mathrm{bio}}(t)}{dt}& =& {\mathbf{s}}_{\mathrm{bio}}^T\cdotp \mathbf{v}(t)\cdotp {c}_{\mathrm{bio}}(t)& \end{array} $$

(4) $$ \begin{array}{lcrr}\frac{d{\mathbf{c}}_{\mathrm{int}}(t)}{dt}& =& {\mathbf{S}}_{\mathrm{int}}\cdotp \mathbf{v}(t)-\mu (t)\cdotp {\mathbf{c}}_{\mathrm{int}}(t)& \end{array} $$

with **c**_ext_ the (*m*_ext_×1) vector of extracellular concentrations [mmol/L], *c*_bio_ the scalar biomass concentration [gDW/L], **S**_ext_ the *m*_ext_ rows of the stoichiometric matrix which correspond to the extracellular metabolites and ${\mathbf{s}}_{\mathrm{bio}}^T$ the row of the stoichiometric matrix which corresponds to the biomass pseudo-metabolite. This system is for the description of concentration evolution in a bioreactor operating in batch-mode, but of course transport terms can be added to make it suitable for fed-batch or continuous operation of bioreactors. It is important to notice that **c**_ext_ and *c*_bio_ are defined per liter of medium, while **c**_int_ is defined per gram of biomass dry weight. As the fluxes are specific, i.e., defined per gram of biomass dry weight, which is more descriptive from a kinetics point of view, the fluxes are multiplied with the biomass concentration in Equations (2) and (3). Again, to fully define this model structure, expressions for all fluxes are needed. These expressions are typically estimated from experimental data.

### Simplifying the multi-scale model: assuming a pseudo steady-state

Because the number of fluxes, and hence the number of expressions which need to be identified and estimated from experimental data, grows quite large for even a medium-scale metabolic reaction network, a simplification is typically made in the form of a *pseudo steady-state assumption*. First, the dilution term in Equation (4) is discarded as it is typically much smaller than the reaction term [7]. Based on the empirical knowledge that the intracellular dynamics are much faster than the extracellular dynamics [8], the pseudo steady-state assumption can be used to simplify the intracellular part of the multi-scale model to the following (*m*_int_×*n*) system of linear equations:

(5) $$ \begin{array}{lcrr}{\mathbf{S}}_{\mathrm{int}}\cdotp \mathbf{v}(t)& =& 0& \end{array} $$

In the majority of metabolic reaction networks, the number of intracellular metabolites is smaller than the number of reactions, making this an underdetermined system of linear equations. The number of degrees of freedom *d* in the system equals the number of unknowns minus the number of independent equations, i.e., *d*=*n*−*rank*(**S**_int_). All solutions to this system can be written as a linear combination of a set of independent fluxes, called the *free fluxes*:

(6) $$ \begin{array}{lcrr}\mathbf{v}(t)& =& \mathbf{K}\cdotp \mathbf{u}(t)& \end{array} $$

with **K** a suitable basis for the null space of **S**_int_ of dimensions (*n*×*d*) and **u** the (*d*×1) vector of free fluxes. By substituting this into the extracellular model, the following simplified model arises:

(7) $$ \begin{array}{lcrr}\frac{d{\mathbf{c}}_{\mathrm{ext}}(t)}{dt}& =& {\mathbf{S}}_{\mathrm{ext}}\cdotp \mathbf{K}\cdotp \mathbf{u}(t)\cdotp {c}_{\mathrm{bio}}(t)& \end{array} $$

(8) $$ \begin{array}{lcrr}\frac{dc_{\mathrm{bio}}(t)}{dt}& =& {\mathbf{s}}_{\mathrm{bio}}^T\cdotp \mathbf{K}\cdotp \mathbf{u}(t)\cdotp {c}_{\mathrm{bio}}(t)& \end{array} $$

Note that to fully define this simplified model, only expressions for the free fluxes need to be identified, resulting in a substantial reduction in experimental and numerical cost.

The resulting model can be written in a more concise way by putting together all concentration variables in the (*m*_ext_+1×1) state vector **x**:

(9) $$ \begin{array}{lcrr}\frac{d\mathbf{x}(t)}{dt}& =& {\mathbf{S}}_{\mathrm{e}}\cdotp \mathbf{K}\cdotp \mathbf{u}(t)\cdotp {\mathbf{q}}_{\mathrm{bio}}^T\cdotp \mathbf{x}(t)& \end{array} $$

with:

(10) $$ \mathbf{x}(t)=\left[\begin{array}{c}{\mathbf{c}}_{\mathrm{ext}}(t)\\ {}{c}_{\mathrm{bio}}(t)\end{array}\right] $$

(11) $$ {\mathbf{S}}_{\mathrm{e}}=\left[\begin{array}{c}{\mathbf{S}}_{\mathrm{e}\mathrm{xt}}\\ {}{\mathbf{s}}_{\mathrm{bio}}^T\end{array}\right] $$

with **S**_e_ of size (*m*_ext_+1×*n*), and ${\mathbf{q}}_{\mathrm{bio}}^T$ a (1×*m*_ext_+1) row vector which selects the last element of **x**, the biomass concentration, i.e., ${\mathbf{q}}_{\mathrm{bio}}^T=\left[0\kern0.60em 0\kern0.60em \dots \kern0.60em 0\kern0.60em 1\right]$. This more concise representation will be used in the remainder of this text.

### Dynamic metabolic flux analysis

The *dynamic metabolic flux analysis* (dMFA) problem now consists of identifying the free flux profiles over time, based on measurements of the states, i.e., the extracellular metabolite concentrations, or the fluxes themselves. This problem can be written as a dynamic input estimation problem using a least-squares objective function:

(12) $$ \begin{array}{l}\underset{\mathbf{u}(t),{\mathbf{x}}_0}{\mathrm{minimize}}\sum_{i=1}^{n_{\mathrm{time}}}\sum_{j=1}^{n_{\mathrm{out}}}{\left(\frac{y_j\left({t}_i\right)-{m}_{ij}}{\sigma_{ij}}\right)}^2\end{array} $$

subject to:

(13) $$ \begin{array}{lcrr}\dot{\mathbf{x}}(t)& =& {\mathbf{S}}_{\mathrm{e}}\cdotp \mathbf{K}\cdotp \mathbf{u}(t)\cdotp {\mathbf{q}}_{\mathrm{bio}}^T\cdotp \mathbf{x}(t)& \end{array} $$

(14) $$ \begin{array}{lcrr}\mathbf{x}(0)& =& {\mathbf{x}}_0& \end{array} $$

(15) $$ \begin{array}{lcrr}\mathbf{y}(t)& =& \mathbf{f}\left(\mathbf{x}(t),\mathbf{u}(t)\right)& \end{array} $$

(16) $$ \begin{array}{lcrr}\mathbf{z}(t)& =& {\mathbf{I}}_{\mathrm{irr}}\cdotp \mathbf{K}\cdotp \mathbf{u}(t)& \end{array} $$

(17) $$ \begin{array}{lcrr}\mathbf{x}(t)& \ge & 0,\mathbf{z}(t)\ge 0& \end{array} $$

with *n*_time_ the total number of time points at which measurements were taken and *n*_out_ the number of outputs of the system. This system includes algebraic states **z**(*t*), which represent the irreversible fluxes. By constraining these states to be positive, the irreversible fluxes are also kept positive. Furthermore, **y**(*t*) is the (*n*_out_×1) vector of outputs of the system, which can be any non-linear function **f** of the states and free fluxes, and *y*_*j*_(*t*_*i*_) is the model output *j* at time *t*_*i*_. The objective function is a weighted sum of least squares, with *m*_*ij*_ and *σ*_*ij*_ respectively the average and the standard deviation for the measurement of output *j* at time point *t*_*i*_, and **x**_0_ is the vector of initial values for the states, which is typically also an optimization variable.

This problem has been treated in literature in different ways. Antoniewicz [9] identifies four approaches for dMFA. (*i*) The first approach [10] divides the experimental time domain in metabolic phases, after which in each phase a classical, static MFA problem is solved, based on averaged measurements of exchange fluxes. This method does not produce time-resolved fluxes. (*ii*) In another approach [11], the measurements themselves are approximated by spline functions, which are then differentiated, resulting in a set of extra flux measurements. These measurements are then used to estimate the (free) fluxes using a series of standard, static MFA problems at different points in time. This approach is easy to use, but presents a number of disadvantages [5]. The most important disadvantage is the fact that every set of data is fitted with its own, independent set of parameters, disregarding the correlation between the different measurements in the estimation process itself. Due to this independent estimation, it is also not possible to use a consistent criterion for assessing the goodness of the fit, as not all data are taken into account in every estimation problem. Furthermore, by representing the dynamic problem as a series of disconnected, static problems, important information on the dynamic nature of the system is lost. Further information loss also occurs when taking derivatives of the spline functions. Also the dynamic, possibilistic framework of Llaneras et al. [12] can be categorized in this class. In this framework, dynamic extracellular concentration measurements are taken into account into a possibilistic MFA strategy by approximating the derivatives of the concentrations. Again, the need for numerical differentiation is an important drawback of also this methodology. (*iii*) Most of these drawbacks have been overcome using the approach described in [5]. In this method, the free fluxes are not estimated as specific fluxes, but are combined with the biomass concentration to non-specific free fluxes. These are then parameterized as piecewise linear functions, which ascertains that the dynamic system can be solved analytically, resulting in a non-dynamic, non-linear parameter estimation problem. This approach, unfortunately, introduces some new drawbacks, i.e., the fact that specific fluxes, which are most descriptive from a biological kinetics point of view, cannot be estimated directly, again resulting in a loss of information when these need to be calculated, the non-smoothness of the flux profiles because of the piecewise linear description, and the fact that the irreversibility constraints on the fluxes are not taken into account. (*iv*) The most recent approach, called *Dynamic Flux Estimation (DFE)* [13], uses power-law or Michaelis-Menten kinetic functions to describe the fluxes, which must be *a priori* postulated. This kinetic information is not yet available for all metabolic reactions in a range of environmental conditions, so an approach which does not need these kinetic functions is preferred. The interested reader is referred to [9] for a review of applications of these four classes of methods for dMFA.

The approach which is presented in this work addresses the disadvantages of previous methods by establishing the true non-linear, dynamic nature of the dMFA problem and using state-of-the-art tools for solving this kind of problems. More specifically, the dynamic optimization problem is solved using (*i*) direct collocation on finite elements to obtain a finite dimensional optimization problem, and (*ii*) automatic differentiation to calculate exact first and second order information. The smoothness of the free flux profiles is ensured by using B-spline functions of second order. B-splines have already been used to discretize the state variables for parameter estimation in biological models [14], but not yet to discretize the fluxes in dMFA models. By using all data at once in the parameter estimation process, the *goodness-of-fit* of the resulting model can be assessed in a consistent way. Furthermore, no knowledge of the kinetics of the different metabolic reactions is needed. However, if this information is available, it can easily be integrated in this methodology in all possible functional forms, including non-linear expressions, as the problem is solved as a non-linear optimization problem.

## Methods

This section is organized in the following way. First, the dMFA problem is transformed into a dynamic parameter estimation problem by use of the B-spline parameterizations. This problem is then further discretized by applying the orthogonal collocation technique, rendering the definition of an NLP subproblem for a fixed number of internal knots in the different spline functions. An adaptive, incremental algorithm to generate a sequence of these subproblems is then defined, in which the number of internal knots is systematically increased, and the experimental horizon is elongated until the full experiment is described. After this, an extension of the algorithm is described in which the **K** matrix is chosen optimally, i.e., in such a way that the *sum of squared errors* (*SSE*) is minimized. This extension makes the algorithm fully integrated, once a network is chosen and measurements are provided. Finally, the determination of the confidence algorithms for the flux estimates is outlined.

### Parameterization of the free fluxes using B-splines

In this novel approach, every free flux is parameterized as a polynomial spline function, based on B-spline basis functions [15]. These B-spline functions are defined by (*i*) the degree *k*, (*ii*) the locations of the *g*+2 so-called knots *t*_0_,*t*_1_,…,*t*_*g*_,*t*_*g*+1_, of which the middle ones are the *g* internal knots, and (*iii*) the *q* control points, or spline parameters **p**_u_. To get a smooth flux profile, i.e., a function with continuous first derivative, the degree of the spline function should be at least two, and for this reason the degree will be fixed to two in this work, i.e., *k*=2. Also, the start and end knots (*t*_0_ and *t*_*g*+1_) are fixed at respectively the start and end times of the experimental horizon under consideration. This leaves three entities to calibrate: the number of internal knots, the internal knot locations and the spline parameters. The basis functions are defined recursively by the Cox-de Boor recursion formula:

(18) $$ {B}_{i,0}(t)=\left\{\begin{array}{cc}1& \kern1em \mathrm{if}\kern0.60em {t}_i\le t\le {t}_{i+1}\\ {}0& \kern1em \mathrm{otherwise}\end{array}\right. $$

(19) $$ {B}_{i,p}(t)=\frac{t-{t}_i}{t_{i+p}-{t}_i}\cdotp {B}_{i,p-1}(t)+\frac{t_{i+p+1}-t}{t_{i+p+1}-{t}_{i+1}}\cdotp {B}_{i+1,p-1}(t) $$

The spline function is then defined as a linear combination of the B-spline basis functions with the spline parameters as coefficients:

(20) $$ \hat{u}(t)=\sum_{i=1}^q{p}_{\mathrm{u},i}\cdotp {B}_{i-1,k}(t) $$

Because of the fact that the vector space of spline functions of degree *k* with *g*+2 knots has dimension *g*+*k*+1, the number of B-spline basis functions and corresponding spline parameters is related to the degree and the number of internal knots as follows:

(21) $$ q=g+k+1 $$

However, based on the recursive definition, only *g*+2−(*k*+1)=*g*−*k*+1 basis functions can be defined from the *g*+2 knots. This means that 2*k* extra knots need to be added to fully define the spline function. This is usually done by adding *k* knots at the beginning and at the end of the knot sequence, equal to the starting and ending knot, respectively [15,16]. For *k*=2, e.g., the total sequence of knots is *t*_0_,*t*_0_,*t*_0_,*t*_1_,…,*t*_*g*_,*t*_*g*+1_,*t*_*g*+1_,*t*_*g*+1_. Based on this total knot vector and the order, the spline functions can be efficiently evaluated using the Cox-de Boor algorithm [17].

**Figure 2:**
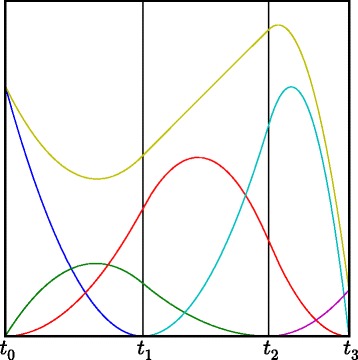
**Example of a second degree B-spline function.** A second degree spline function with two internal knots (in yellow), along with the B-spline functions that generate it (in blue, green, red, cyan and pink). In this figure, the B-spline basis functions are already multiplied with their corresponding spline parameter, so the spline function is obtained by summing the 5 basis functions at each point in time.

A second degree spline function with two internal knots is shown in Figure 2, along with the B-spline basis functions that generate the spline. In this figure, the basis functions are already multiplied with their corresponding spline parameter. It is clear that the different basis functions are only non-zero in a part of the interval, i.e., the spline parameters only influence a part of the final spline function. This property is called *local support*. The location of the internal knots is also important, because, when more knots are situated in a specific region, there is a higher flexibility in that specific region, enabling the function to have a more exotic shape. As will also be seen in the Results and discussion section, the internal knots will flock together in regions in which there is a high curvature, and will move away from flat regions.

In the final sequential algorithm, three main operations on the splines are used. The first operation is *constraining a knot to the specific measurement interval it is in*. This is done to prevent knots from straying too far from their initial optimal location. The second operation is *inserting a knot at the end of a specified time frame*. In this operation, knot insertion, a feature inherent to B-splines, is used to insert a knot without changing the spline profile. This way, the next optimization can be started from a good initial guess, with an extra knot inserted. This operation also takes into account the bounds which were placed on previously added knots, i.e., the knot is inserted in the time frame at the end, where no knot has yet been inserted. The last operation *prolongates the splines*, which only changes the ending knot to the new value. This means that the spline profile is slightly changed, because the spline parameters stay the same, but is still close to the previous profile.

Each free flux is represented by its own spline function, and thus has its own set of internal knot locations and spline parameters. For ease of notation, the spline parameters for the different free fluxes will be concatenated into three vectors:

(22) $$ \mathbf{g}=\left[\begin{array}{c}{g}_1\\ {}\vdots \\ {}{g}_d\end{array}\right] $$

(23) $$ {\mathbf{t}}_{\mathrm{knot}}=\left[\begin{array}{c}{\mathbf{t}}_{\mathrm{knot},1}\\ {}\vdots \\ {}{\mathbf{t}}_{\mathrm{knot},d}\end{array}\right] $$

(24) $$ {\mathbf{p}}_{\mathrm{u}}=\left[\begin{array}{c}{\mathbf{p}}_{\mathrm{u},1}\\ {}\vdots \\ {}{\mathbf{p}}_{\mathrm{u},d}\end{array}\right] $$

The vector **g** contains the number of internal knots for the *d* free fluxes, which are integer variables. The vector **t**_knot_ contains the internal knot locations for all free fluxes, i.e., this vector contains *n*_g_ elements, with ${n}_{\mathrm{g}}=\sum_{i=1}^d{g}_i$. The last vector **p**_u_ contains all spline parameters or control points for all free fluxes. The number of elements in this vector equals *n*_g_+*d*·(*k*+1). In total, there are *d* integer parameters, the numbers of internal knots, and 2·*n*_g_+*d*·(*k*+1) continuous parameters, the knot locations and spline parameters, to estimate.

### Formulation of the dynamic estimation problem

The parameters in **g** directly control the number of the other parameters. As the least squares objective will keep on decreasing with increasing number of parameters, the optimal **g** will contain infinity values at all elements, rendering a perfect fit. For this reason, **g** is not added as an optimization variable in the optimization problem. The estimation of these values will be addressed later on. Using the B-spline flux parametrizations, the input estimation problem (Equation (12)) is reformulated as a dynamic parameter estimation problem:

(25) $$ \begin{array}{l}\underset{{\mathbf{p}}_{\mathrm{u}},{\mathbf{t}}_{\mathrm{knot}},{\mathbf{x}}_0}{\mathrm{minimize}}\sum_{i=1}^{n_{\mathrm{time}}}\sum_{j=1}^{n_{\mathrm{out}}}{\left(\frac{y_j\left({t}_i\right)-{m}_{ij}}{\sigma_{ij}}\right)}^2\end{array} $$

subject to:

(26) $$ \begin{array}{lcrr}\dot{\mathbf{x}}(t)& =& {\mathbf{S}}_{\mathrm{e}}\cdotp \mathbf{K}\cdotp \widehat{\mathbf{u}}(t)\cdotp {\mathbf{q}}_{\mathrm{bio}}^T\cdotp \mathbf{x}(t)& \end{array} $$

(27) $$ \begin{array}{lcrr}\mathbf{x}(0)& =& {\mathbf{x}}_0& \end{array} $$

(28) $$ \begin{array}{lcrr}\mathbf{y}(t)& =& \mathbf{f}\left(\mathbf{x}(t),\widehat{\mathbf{u}}(t)\right)& \end{array} $$

(29) $$ \begin{array}{lcrr}\mathbf{z}(t)& =& {\mathbf{I}}_{\mathrm{irr}}\cdotp \mathbf{K}\cdotp \widehat{\mathbf{u}}(t)& \end{array} $$

(30) $$ \begin{array}{lcrr}\mathbf{x}(t)& \ge & 0,\mathbf{z}(t)\ge 0& \end{array} $$

### Discretization of the dynamic parameter estimation problem using collocation

The resulting dynamic optimization problem must be discretized in some way to be able to solve it [18]. In this work, direct collocation on finite elements was chosen [19]. For a full overview on the direct collocation method, the reader is referred to [18]. For the methodology in this work, cubic Lagrange polynomials were chosen, with collocation points situated at the Radau roots. The finite element borders were chosen at the time points of the measurements.

The direct collocation method turns the differential and algebraic states into discrete, continuous variables **p**_x_ and **p**_z_, respectively, and the dynamic system into two sets of equality constraints: (*i*) the collocation constraints **h**_coll_, which make sure that the polynomials satisfy the dynamic system exactly at the collocation points, and (*ii*) the continuity constraints **h**_cont_, which ensure the continuity of the Lagrange polynomials over the finite element borders. After discretizing the system of ODEs, the dynamic parameter estimation problem turns into the following non-linear programming problem (NLP):

(31) $$ \begin{array}{l}\underset{{\mathbf{p}}_{\mathrm{x}},{\mathbf{p}}_{\mathrm{z}},{\mathbf{p}}_{\mathrm{u}},{\mathbf{t}}_{\mathrm{knot}},{\mathbf{x}}_0}{\mathrm{minimize}}\sum_{i=1}^{n_{\mathrm{time}}}\sum_{j=1}^{n_{\mathrm{out}}}{\left(\frac{y_j\left({t}_i\right)-{m}_{ij}}{\sigma_{ij}}\right)}^2\end{array} $$

subject to:

(32) $$ \begin{array}{lcrr}{\mathbf{h}}_{\mathrm{coll}}\left({\mathbf{p}}_{\mathrm{x}},{\mathbf{p}}_{\mathrm{z}},{\mathbf{p}}_{\mathrm{u}},{\mathbf{t}}_{\mathrm{knot}}\right)& =& 0& \end{array} $$

(33) $$ \begin{array}{lcrr}{\mathbf{h}}_{\mathrm{cont}}\left({\mathbf{p}}_{\mathrm{x}},{\mathbf{p}}_{\mathrm{z}}\right)& =& 0& \end{array} $$

(34) $$ \begin{array}{lcrr}\widehat{\mathbf{x}}(0)& =& {\mathbf{x}}_0& \end{array} $$

(35) $$ \begin{array}{lcrr}\mathbf{y}(t)& =& \mathbf{f}\left(\widehat{\mathbf{x}}(t),\widehat{\mathbf{u}}(t)\right)& \end{array} $$

(36) $$ \begin{array}{lcrr}{\mathbf{p}}_{\mathrm{x}}& \ge & 0,{\mathbf{p}}_{\mathrm{z}}\ge 0& \end{array} $$

with **p**_x_ the collocation variables, including the initial values for the states.

If the spline degree *k* and the Lagrange polynomial degree are fixed to 2 and 3 respectively, and the number of finite elements is taken as *n*_time_−1, as the finite elements are situated between the measurement time points, the dimensions of this problem depend on the total number of internal knots *n*_g_, the number of time points *n*_time_ and the characteristics of the network. These dimensions are given in Table 1.

**Table 1:** **Dimensions of the resulting non-linear programming problems**

**Variables**	
Differential state variables **p**_x_	4·(*n*_time_−1)·(*m*_ext_+1)
Algebraic state variables **p**_z_	4·(*n*_time_−1)·*n*_irr_
Spline parameters **p**_u_	*n*_g_+*d*·(*k*+1)
Internal knot locations **t**_knot_	*n*_g_
Initial values **x**_0_	*m*_ext_+1
**K** matrix values	*n*·*d*
**Equality constraints**	
Differential state collocation constraints	3·(*n*_time_−1)·(*m*_ext_+1)
Differential state continuity constraints	(*n*_time_−2)·(*m*_ext_+1)
Algebraic state collocation constraints	(3·(*n*_time_−1)+1)·*n*_irr_
Algebraic state continuity constraints	(*n*_time_−2)·(*n*_irr_)
Initial value constraints	*m*_ext_+1
**K** null space constraints	*m*_int_·*d*
**K** orthogonality constraints	$\frac{d\cdotp \left(d+1\right)}{2}$

The resulting NLP (31) is solved using the interior-point optimization routine IPOPT [20]. Gradient, Jacobians and Hessian are generated exactly using automatic differentiation with CasADi [21].

### Adaptive incremental free flux estimation

The number of internal knots directly controls the number of the other parameters. As the least squares objective will keep on decreasing with increasing number of parameters, the optimal number of knots will be infinity, rendering a perfect fit of the measurement noise instead of the trend. For this reason, a mechanism to prevent overfitting has to be used. Furthermore, although the polynomial spline functions are linear functions of the spline parameters, the system of ODEs is non-linear, and the splines are also non-linear in the knot locations. These non-linearities in the constraints lead to local minima in the optimization problem. To address these issues, a systematic, incremental strategy for estimating the free flux parameters and knot locations has been devised, based on the Akaike model discrimination criterion (*AIC*). This criterion [22] is frequently used to discriminate between different model structures which can describe the same phenomenon. It takes into account both the model error, i.e., the least squares error, and the number of parameters needed to describe the data. It has been applied successfully for model discrimination in both linear and non-linear models for biological systems (see, e.g., [23]). In this work, the corrected *AIC* criterion for small sample sizes (*AIC*_c_) is used, as this is more suited in cases where the number of measurements is close to the number of parameters:

(37) $$ \begin{array}{lcrr}{AIC}_{\mathrm{c}}& =& f+2\cdotp {n}_{\mathrm{p}}+\frac{2\cdotp {n}_{\mathrm{p}}\cdotp \left({n}_{\mathrm{p}}+1\right)}{n_{\mathrm{meas}}-{n}_{\mathrm{p}}-1}& \end{array} $$

with *f* the weighted least squares error, as defined in Equation (31), and *n*_meas_ the total number of measurements, i.e., *n*_time_·*n*_out_. From this definition it is clear that:

(38) $$ {n}_{\mathrm{meas}}\kern0.60em \ge \kern0.60em {n}_{\mathrm{p}}+2 $$

as otherwise the denominator can become zero or negative. In the remainder of this text, *AIC*_c_ is indicated just as *AIC*, for simplicity.

**Figure 3:**
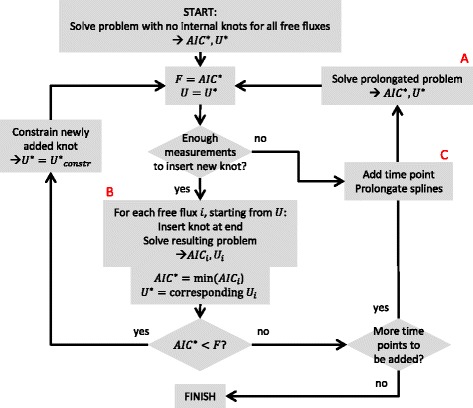
**Incremental flux estimation algorithm.** A schematic representation of the algorithm for the incremental flux estimation. The steps marked with a red **A**, **B** and **C** correspond to the steps in Figure 7.

An algorithmic description of the methodology is given in Figure 3. The method is started by estimating splines without knots, i.e., only second degree polynomials, on a reduced dataset, i.e., the first *l* timepoints, where *l* is the number of timepoints needed to make sure that the denominator of Equation (37) is strictly positive:

(39) $$ l\cdotp {n}_{\mathrm{out}}\kern0.60em \ge \kern0.60em 3\cdotp d+{m}_{\mathrm{ext}}+3 $$

After selecting the correct number of timepoints, the polynomials are fitted and the optimal *AIC* value is saved as *F*, together with the optimal splines *U*. Then, it is checked if a new knot can be inserted based on the number of measurements available at this point. If so, *d* optimization problems are generated, in which every time one knot is inserted into one free flux spline at a time. The three problems are solved, and the minimum *AIC* value over these problems is saved as *AIC*^∗^, along with the corresponding optimal splines *U*^∗^, in which there is now one knot in one of the splines. Now, two possibilities arise. If *AIC*^∗^ is smaller than *F*, a new, better solution is found, and another knot can be added using the same steps just described. If *AIC*^∗^ is higher than *F*, however, the old optimum was better than the new one, and so the old one is kept. At this point, an optimal solution for this number of time points has been found, and a new time point can be added if there is still one left to be added. After adding the time point, the splines are prolongated, and the prolongated problem is solved to get the new starting values for *F* and *U* for the next iteration. Once all time points have been added, *U* contains the final, optimal set of free flux profiles for the specified dataset.

Although the Akaike criterion can compare different models, it cannot assess the absolute goodness-of-fit. To this end, a *χ*^2^-test is also executed on the resulting model [24]. The resulting model is accepted if the variance weighted sum of squared errors is smaller than the critical *χ*^2^-value for the specified confidence level (95%) and number of degrees of freedom, which is the number of measurements minus the number of independent parameters.

This methodology ensures that each optimization problem is initialized with an excellent initial guess for the knot locations and spline parameters, rendering shorter optimization times and convergence to at least a decent local minimum in each iteration.

### Choice of the null space basis **K**

The only degree of freedom left at this point is the choice of the basis **K** for the null space of **S**_int_. The choice of this basis defines which fluxes are used as free fluxes, and as these are the profiles that are estimated, this choice can have significant consequences concerning the final fit of the model. Free fluxes with a large curvature in their profile need more parameters to be successfully estimated. There are three options: (*i*) a fixed rational basis, (*ii*) a fixed orthonormal basis, or (*iii*) an optimal orthonormal basis. A rational basis is derived from the reduced row echelon form of **S**_int_. In this form, *d* fluxes are chosen as free fluxes, and the other ones are linear combinations of these free fluxes. The rational basis has the advantage that the free fluxes are easy to interpret, but they are probably not the best choice considering goodness-of-fit. An orthonormal basis is typically derived from the singular value decomposition of **S**_int_. In this case, the free fluxes are themselves linear combinations of the fluxes, rendering free fluxes which cannot be easily interpreted, although the set of all fluxes is still easily calculated from the free fluxes. This basis is numerically preferable, but still not optimal considering goodness-of-fit. As a last possibility, the basis can be optimized during the estimation of the fluxes. To do this, the values in the **K** matrix are added as optimization variables, and two matrix constraints are added, one that defines **K** to be a null space of **S**_int_, and another one which constrains **K** to be orthonormal:

(40) $$ \begin{array}{lcrr}{\mathbf{S}}_{\mathrm{int}}\cdotp \mathbf{K}& =& 0& \end{array} $$

(41) $$ \begin{array}{lcrr}{\mathbf{K}}^T\cdotp \mathbf{K}-\mathbf{I}& =& 0& \end{array} $$

with **I** a (*d*×*d*) identity matrix. In this last constraint, only the diagonal and one of the two off-diagonal parts are independent, since **K**^*T*^·**K** is symmetric. In total, *n*·*d* variables are added (all elements of **K**), along with *m*_int_·*d* null space constraints and $\frac{d\cdotp \left(d+1\right)}{2}$ orthogonality constraints, rendering $\frac{d\cdotp \left(d-1\right)}{2}$ extra degrees of freedom for the optimization.

### Formulation of the non-linear estimation problem with an optimal **K**

When using the optimal basis, the optimization problem looks like this:

(42) $$ \begin{array}{l}\underset{{\mathbf{p}}_{\mathrm{x}},{\mathbf{p}}_{\mathrm{z}},{\mathbf{p}}_{\mathrm{u}},{\mathbf{t}}_{\mathrm{knot}},{\mathbf{x}}_0,\mathbf{K}}{\mathrm{minimize}}\sum_{i=1}^{n_{\mathrm{time}}}\sum_{j=1}^{n_{\mathrm{out}}}{\left(\frac{y_j\left({t}_i\right)-{m}_{ij}}{\sigma_{ij}}\right)}^2\end{array} $$

subject to:

(43) $$ \begin{array}{lcrr}{\mathbf{h}}_{\mathrm{coll}}\left({\mathbf{p}}_{\mathrm{x}},{\mathbf{p}}_{\mathrm{z}},{\mathbf{p}}_{\mathrm{u}},{\mathbf{t}}_{\mathrm{knot}},\mathbf{K}\right)& =& 0& \end{array} $$

(44) $$ \begin{array}{lcrr}{\mathbf{h}}_{\mathrm{cont}}\left({\mathbf{p}}_{\mathrm{x}},{\mathbf{p}}_{\mathrm{z}}\right)& =& 0& \end{array} $$

(45) $$ \begin{array}{lcrr}\widehat{\mathbf{x}}(0)& =& {\mathbf{x}}_0& \end{array} $$

(46) $$ \begin{array}{lcrr}\mathbf{y}(t)& =& \mathbf{f}\left(\widehat{\mathbf{x}}(t),\widehat{\mathbf{u}}(t)\right)& \end{array} $$

(47) $$ \begin{array}{lcrr}{\mathbf{p}}_{\mathrm{x}}& \ge & 0,{\mathbf{p}}_{\mathrm{z}}\ge 0& \end{array} $$

(48) $$ \begin{array}{lcrr}{\mathbf{S}}_{\mathrm{int}}\cdotp \mathbf{K}& =& 0& \end{array} $$

(49) $$ \begin{array}{lcrr}{\mathbf{K}}^T\cdotp \mathbf{K}-\mathbf{I}& =& 0& \end{array} $$

### Determination of confidence bounds on the estimated flux profiles

After the optimal model is estimated, uncertainty of the parameters and free flux profiles is estimated using a Monte Carlo bootstrapping methodology [25]. The Monte Carlo approach is used frequently in MFA studies [26]. In this work, the method is used because of the non-linearity and constraints in the optimization problem. In this case, the Fisher information approach can give highly different confidence intervals because of these non-linearities and bounds. Based on the assumption of normally distributed measurements with known variances, 1000 sets of measurements were sampled from these distributions and for each set of measurements, parameters were estimated, resulting in 1000 sets of parameter values and 1000 free flux profiles. 95% confidence intervals were generated by sorting these parameters and taking the 2.5^th^ and 97.5^th^ percentiles as respectively lower and upper confidence bounds.

## Results and discussion

This results section is structured in the following way. First, the small-scale case study is presented, along with the network, simulated measurements and reference fluxes used to simulate these measurements. For the small-scale network, different cases with and without measurement noise, and with different **K** matrices, are introduced. Then, a detailed description of the iterations made by the algorithm for one case is given, to further clarify the operation. After this, the results of all cases in the small-scale case study are shown and discussed, highlighting the most important features of this methodology. Then, the medium-scale case study is treated, again by showing the network, simulated measurements and reference fluxes. Finally, the results for this case study are presented, with both the fixed and optimal (free) **K** matrices, along with an analysis of the computational complexity for both case studies.

### Description of the small-scale case study

**Figure 4:**
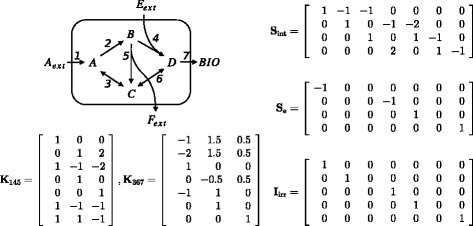
**Case study network and corresponding matrices.** Metabolic reaction network for the case study (top left), along with the intracellular and combined extracellular and biomass stoichiometric matrices and irreversibility matrix (right), and the null space basis matrices corresponding to the cases with free fluxes 1, 4 and 5, and free fluxes 3, 6 and 7 (bottom left).

The network is shown in Figure 4, along with the corresponding **S**_int_, **S**_e_ and **I**_irr_ matrices. It consists of 3 extracellular metabolites and biomass, 4 intracellular metabolites and 7 fluxes. Thus, the number of free fluxes is 3. For the simulation of the measurements, these were chosen as flux 1, 4 and 5. Measurements were simulated by choosing reference profiles for these three fluxes, and simulating the states using the dynamic system.

(50) $$ \begin{array}{lcrr}{u}_{1,\mathrm{r}\mathrm{e}\mathrm{f}}& =& \frac{c_{\mathrm{Aext}}}{1.5+{c}_{\mathrm{Aext}}}& \end{array} $$

(51) $$ \begin{array}{lcrr}{u}_{4,\mathrm{r}\mathrm{e}\mathrm{f}}& =& 0.2\cdotp \frac{c_{\mathrm{Eext}}}{3+{c}_{\mathrm{Eext}}}& \end{array} $$

(52) $$ \begin{array}{lcrr}{u}_{5,\mathrm{r}\mathrm{e}\mathrm{f}}& =& \frac{1}{1+{c}_{\mathrm{Fext}}}& \end{array} $$

**Figure 5:**
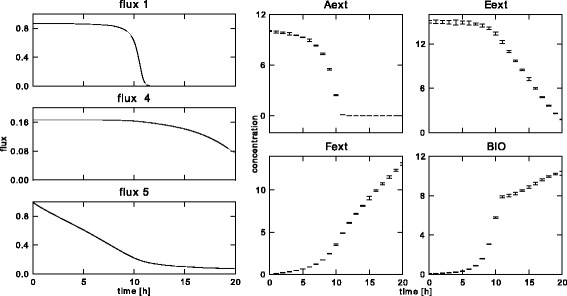
**Reference free flux profiles and simulated measurements for the realistic noise setting.** The profiles for the free fluxes (left) that were used to simulate the state measurements (right). The measurements in this figure correspond to the realistic noise setting, with different variances on all measurement points.

The starting values for the 4 states were chosen at 10, 15, 0 and 0.1, respectively. Two sets of measurements were generated, both at 21 equidistant points in time between 0 and 20 for all 4 states, rendering both 84 measurements in total: (*i*) one set with normal noise with a variance of 10^−8^, to test the capabilities of the algorithm without measurement error, and (*ii*) one set with a different, realistic variance for every measurement, to test the capabilities in a more realistic setting. A value for the variance of every measurement is necessary for the algorithm, because of the use of the variance-weighted sum of squared errors in the *AIC* criterion. For that reason, the variance in the first case was not set at 0, but at a very low value. The reference profiles and the simulated data based on these profiles for the realistic noise realization are shown in Figure 5.

The methodology was executed for 4 different **K** matrices: (*i*) the one corresponding to free fluxes 1, 4 and 5, i.e., the same one as was used to generate the simulated measurements, (*ii*) the one corresponding to free fluxes 3, 6 and 7, of which free fluxes 3 and 6 are reversible, as opposed to the first case where all free fluxes are irreversible, (*iii*) an orthonormal basis obtained through the Matlab command *null*(**S**_int_), and (*iv*) a variable basis which is optimized using the additional constraints as described before. These cases are referred to as *145*, *367*, *orthonormal* and *optimal*, respectively. In total, the algorithm was run 8 times: 4 times for the different cases with low noise, and 4 times for the different cases with the realistic noise realization.

All reactions, all stoichiometric matrices, the irreversibility matrix, the null space basis matrices, and the simulated measurements and measurement variances for this case study are given in the Additional files 1, 2, 3, 4, 5, 6, 7, 8, 9, 10, 11 and 12.

### Description of the algorithm iterations

**Figure 6:**
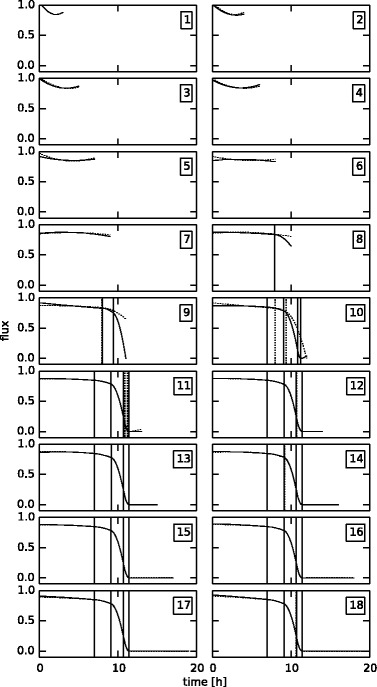
**Intermediate flux profiles for the** ***145*** **case.** Overview of the intermediate flux profiles during the iterations of the algorithm for the *145* case with realistic noise, for free flux 1. The dashed lines are at the beginning of the iteration, the solid lines are at the end of the iteration, before the prolongation step. The iteration number is shown in the top right of each plot.

**Figure 7:**
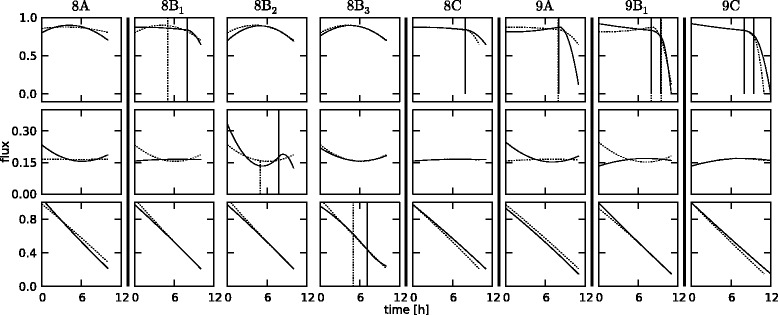
**Detail of iterations 8 and 9 for the** ***145*** **case.** Detailed view of the key steps in iteration 8 and 9 for all three free fluxes (from top to bottom), for the *145* case, with realistic noise. The dashed lines are before optimization, the solid lines after optimization. The A, B and C steps correspond to the steps highlighted in the algorithm in Figure 3.

To validate and clarify the operation of the proposed algorithm, the key iterations for the case with free fluxes 1, 4 and 5 are described in more detail. An overview of the different iterations is given in Table 2 and the profiles for free flux 1 before and after all iterations are depicted in Figure 6. Also, a detailed description of the steps in iteration 8 and 9 for all free fluxes is given in Figure 7.

**Table 2:** **Overview of the iterations done by the incremental flux estimation algorithm for the** ***145*** **case**

**Iter. no.**	***n***_**time**_	***n***_**p**_	***AIC*** ** before insertion**	**Min.** ***AIC***	**Comment**
1	4	13	208.0	—	No new knot possible
2	5	13	86.7	150.0	No better minimum
...	...	...	...	...	...
7	10	13	41.0	50.3	No better minimum
8	11	15	58.6	48.0	Knot inserted in flux 1, interval 7
		15	48.0	57.9	No better minimum
9	12	17	88.2	58.4	Knot inserted in flux 1, interval 9
		17	58.4	65.5	No better minimum
10	13	19	1562.6	1400.7	Knot inserted in flux 1, interval 10
		21	1400.7	100.5	Knot inserted in flux 1, interval 11
		23	100.5	85.8	Knot inserted in flux 3, interval 8
		23	85.8	100.4	No better minimum
11	14	23	81.1	93.8	No better minimum
12	15	23	78.2	89.2	No better minimum
13	16	23	78.8	85.5	No better minimum
14	17	25	92.0	82.4	Knot inserted in flux 3, interval 12
		25	82.4	92.3	No better minimum
15	18	25	80.1	88.9	No better minimum
...	...	...	...	...	...
18	21	25	79.9	84.1	No better minimum

For this network, *n*_out_=4 and *d*=3, so the number of time points needed to start is 4, based on Equation (39). In the first iteration, basically three second degree polynomials are fitted for each of the three fluxes. At this point it is not yet possible to insert a knot because after insertion the number of parameters would be 11, and Equation (38) would not be satisfied anymore. So a new time point is added at the end, and the problem is solved again for this extended dataset. Now, it is possible to insert a knot. Three subproblems are generated, one for each flux spline in which a knot is inserted. The minimum *AIC* for these subproblems (150.0) is however bigger than the previous one (86.7), so no new minimum is found, and the dataset is extended again. This same pattern goes on until iteration 8, which is shown in more detail in Figure 7. In step 8*A*, the prolongated problem of the previous iteration is solved, giving the base value for *F* (58.6), and the starting set of free flux profiles *U* for this iteration. The dashed lines indicate the profiles before optimization, i.e., after the prolongation step of iteration 7, and the solid lines are the profiles after optimization. Step 8*B* consists of three optimizations, one for each free flux. In step 8*B*_1_, a knot is inserted into the spline corresponding to free flux 1, which, as is shown by the dashed lines, does not change the profile of this flux. The profiles are then used as good starting values for the optimization, after which again the solid profiles result. The optimal location of this knot is 7.94. This same procedure is repeated in steps 8*B*_2_ and 8*B*_3_, with insertion in free flux 2 and 3 respectively. After this, the minimum *AIC* value over steps 8*B*_1_, 8*B*_2_ and 8*B*_3_ is collected (48.0), along with the corresponding profiles. In this case, the minimum is found when inserting a knot in free flux 1. Since the minimum *AIC* value is also lower than *F*, the profiles from step 8*B*_1_ form the starting values for a new round of insertions, after a constraint has been added that constrains the location of the newly added knot to its corresponding measurement interval, in this case between 7.0 and 8.0. This new round of insertions does not yield a better minimum (57.9). This means the profiles after step 8*B*_1_ are the best possible in this iteration, and these profiles are prolongated in step 8*C*. As can be seen in the figure, the prolongation changes the profiles slightly, but they are still good starting values for the base optimization of iteration 9 (step 9*A*), yielding an *F* value of 88.2. Because of the knot that is already present in step 9*B*_1_, the new knot for free flux 1 is inserted after time 8.0. The new minimum (58.4) is again found after insertion in free flux 1, and a second insertion in this iteration does not result in an improvement (65.5), so the profiles after step 9*B*_1_ are prolongated in step 9*C*. The algorithm ends in iteration 18, because the full dataset is used at that point, rendering the optimal flux profiles for the full experiment.

The fact that this procedure results in a good sequence of starting values for the different optimization problems is clearly shown in Figure 6. The dashed lines are again the profiles before each iteration, the solid lines are the profiles after the iteration. Because of the high degree of non-linearity resulting from the free knot locations, these good starting values are essential for the efficient estimation of the fluxes.

### Results in the low noise setting

**Figure 8:**
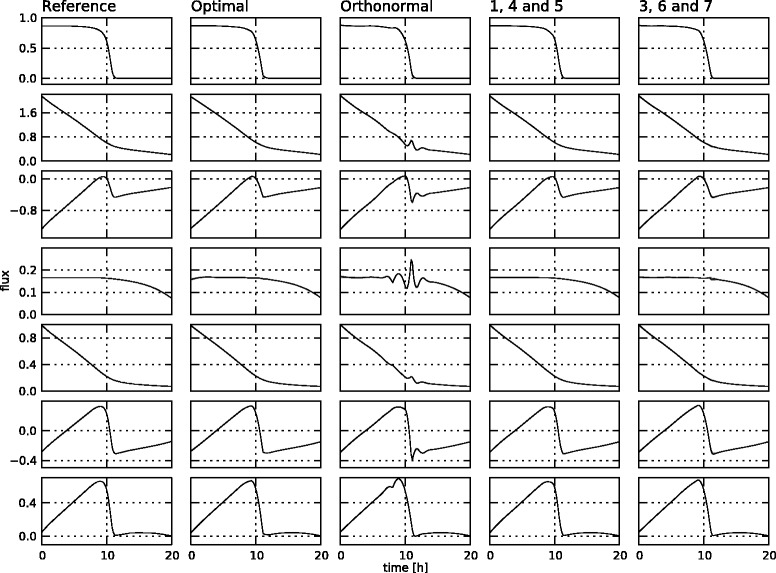
**Estimated flux profiles for the low noise setting.** The reference flux profiles (left column) and estimated flux profiles for the different cases in the low noise setting: the case with optimized **K** matrix (second column), the case with the orthonormal **K** matrix (third column), the *145* case (fourth column), and the *367* case (right column). The profiles are for fluxes 1 through 7 from top to bottom.

**Figure 9:**
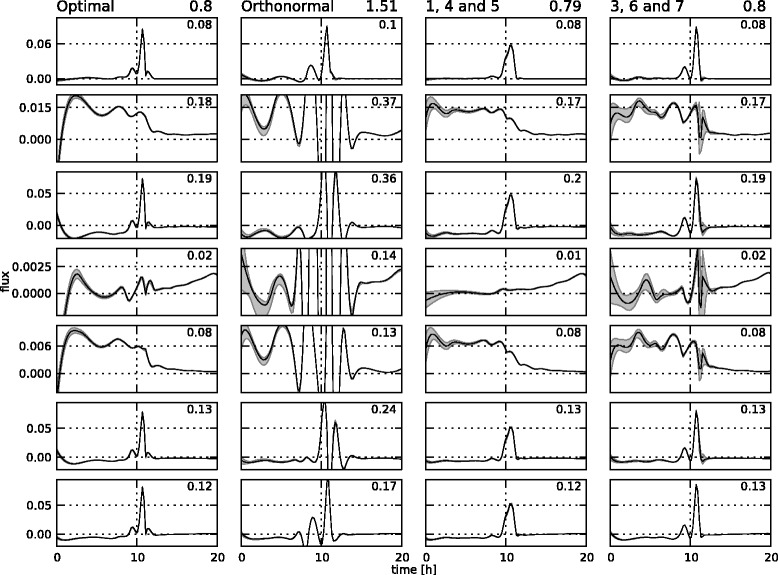
**Absolute deviation of the flux profiles from the reference for the low noise setting.** The deviation of the estimated profiles from the reference for the different cases in the low noise setting: the case with optimized **K** matrix (first column), the case with the orthonormal **K** matrix (second column), the *145* case (third column), and the *367* case (right column). The profiles are for fluxes 1 through 7 from top to bottom. The number on the top right of each graph is the integral of the absolute value of each deviation profile. These numbers are summed for each case at the top of the column, next to the title.

The estimated fluxes for the different choices of the null space basis **K** in the setting with a very small amount of noise are displayed in Figure 8, along with the reference profiles. The absolute error between the estimated profiles and the reference profiles are depicted in Figure 9. This figure also contains the numeric integral of the absolute value of the profiles, as a quantitative way to compare the different alternatives. The sum of all these values is given in the top of the column.

These figures indicate that, except in the *orthonormal* case, the methodology gives an accurate representation of the fluxes in the theoretically optimal low noise setting. The big differences with the reference profiles in the *orthonormal* case are mainly due to numerical problems, since in this low noise setting, a lot of knots are inserted (about 10 per free flux), resulting in harder optimization problems and longer optimization times. Although the other methods for the choice of **K** can cope with these difficulties, this is apparently not the case for the *orthonormal* method. The integral numbers for the other three methods are very close, so there is no clear difference between them in this low noise setting.

### Results for the realistic noise realization

**Figure 10:**
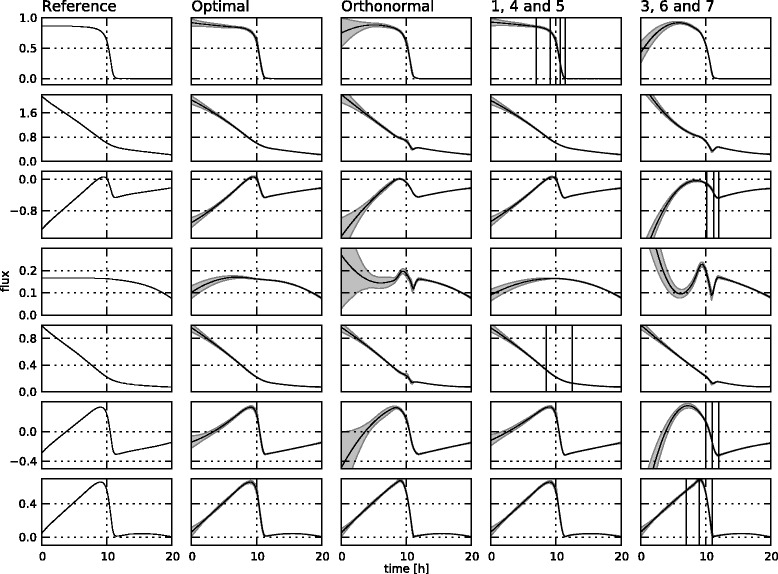
**Estimated flux profiles for the realistic noise setting.** The reference flux profiles (left column) and estimated flux profiles for the different cases in the realistic noise setting: the case with optimized **K** matrix (second column), the case with the orthonormal **K** matrix (third column), the *145* case (fourth column), and the *367* case (right column). The profiles are for fluxes 1 through 7 from top to bottom.

**Figure 11:**
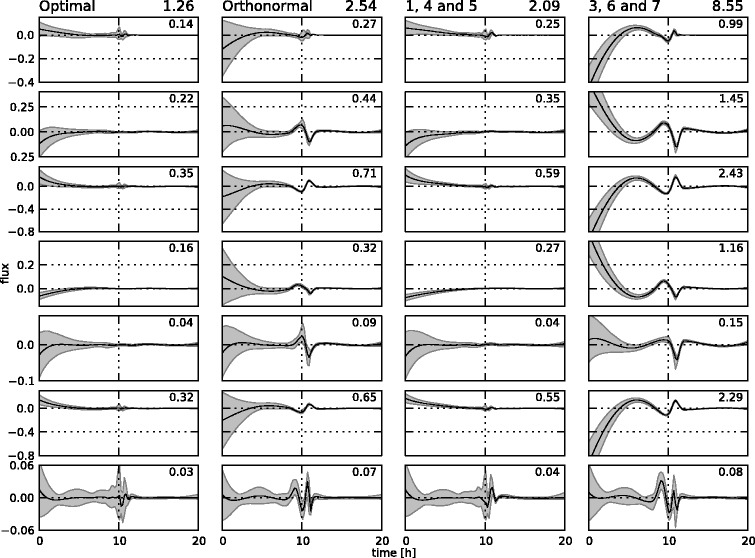
**Absolute deviation of the flux profiles from the reference for the realistic noise setting.** The deviation of the estimated profiles from the reference for the different cases in the realistic noise setting: the case with optimized **K** matrix (first column), the case with the orthonormal **K** matrix (second column), the *145* case (third column), and the *367* case (right column). The profiles are for fluxes 1 through 7 from top to bottom. The number on the top right of each graph is the integral of the absolute value of each deviation profile. These numbers are summed for each case at the top of the column, next to the title.

**Figure 12:**
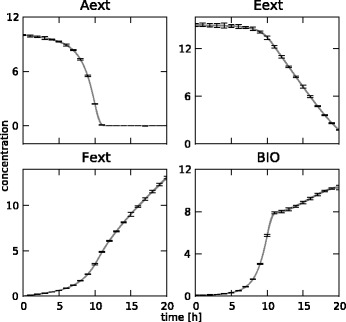
**Estimated state trajectories.** The estimated state trajectories for the *145* case with realistic noise.

The estimated fluxes based on the different choices for the null space basis **K** for the realistic noise setting are displayed in Figure 10, next to the reference profiles. Again, the absolute errors between these estimated profiles and the reference profiles are given in Figure 11, along with the integral differences for each profile, and their sum per case. The state trajectories for the different cases are very similar, so these are only shown for the *145* case in Figure 12. In Table 3, the knot locations and goodness-of-fit values for the different cases are summarized. It is important to note that all estimations are approximations, as the real kinetic law for the fluxes is not known to the estimation procedure. A few observations can be made.

**Table 3:** **Results for the different cases: the intervals where knots are inserted for the three free fluxes, values for** ***SSE*** **and** ***AIC*** **and the number of parameters**

**Choice of K**	**Knot intervals for free flux**			***SSE***	***AIC***	***n***_**p**_
	**1**	**2**	**3**			
1, 4 and 5	7, 9, 10, 11	—	8, 12	7.49	79.9	25
3, 6 and 7	10, 11, 12	10, 11, 12	7, 8, 10, 11	49.50	160.4	33
Orthonormal	8, 11, 12	9, 10, 11	7, 8, 10, 11	14.41	125.3	33
Optimal	7, 9, 10, 11	—	8, 12	1.33	86.9	28

First of all, it is clear that the algorithm puts the knots in locations where they are most needed. In the *145* case, four knots are chosen for flux 1, which exhibits the highest degree of curvature, two knots for flux 5, and no knots for flux 4, which has the flattest profile. Fluxes 3, 6 and 7, on the other hand, all exhibit profiles with a much higher degree of curvature, resulting in a higher total number of knots for the *367* case. Both the *SSE* and the number of parameters are higher for this case, resulting in a higher *AIC* value. Based on these observations, a possible strategy for choosing the basis **K** could be to choose free fluxes with a “flat” profile, i.e., with a low degree of curvature. This is in practice not feasible as the flux profiles are of course not known beforehand. A possible solution would be to choose a basis, estimate the fluxes, and choose a new basis based on the results of the estimation. For this to work, one would need a way of quantifying the degree of curvature of a function, which is not trivial. It would also be computationally very demanding, as the full estimation procedure would have to be repeated a number of times.

The choice of a random orthonormal basis is also clearly not satisfactory. For this case, the knot locations cannot be displayed anymore on the flux profiles, as the free fluxes are in this case linear combinations of the different fluxes. In the final, *optimal* case, from the set of possible orthonormal bases, the one which minimizes the goodness-of-fit is chosen during the course of the algorithm, introducing a minimal number of extra parameters for the optimization. The estimated profiles for this case, as well as for the *145* case, accurately resemble the reference profiles, although the integral numbers indicate that the *optimal* case is slightly better. In the *optimal* case, however, three extra parameters are introduced, resulting in a lower *SSE*, but a slightly higher *AIC* value and computational time. This is only a small penalty, though, not outweighing the benefits of having an accurate estimate using only one run of the algorithm, and the fact that the algorithm is completely contained, as the user does not have to make any choices once the measurements and the details of the network are given. In this simulated case study, the results for the *145* case are probably better because the same basis was used to simulate the measurements. In a real-life setting, though, there is no generating set of free fluxes, and a choice regarding this set of free fluxes cannot be made. Thus, the addition of the determination of the optimal **K** basis is a welcome addition in real-life settings, as the optimal basis is always found, without any choices required by the user. The operation of the algorithm in optimal **K** mode thus makes the algorithm fully contained.

### Description of the medium-scale case study

The medium-scale network was adapted from [5], and all reactions are available in the supplementary data of that work. A few alterations were made, though. The pseudo-reaction of the glucose feed to the glucose in the medium (reaction 59 in [5]), was removed, as this flux was considered constant and thus does not need to be estimated. Furthermore, reaction 41 in [5] (42 in Additional file 13), threonine to glycine, was set to be reversible, as otherwise no biomass could be formed, and also reaction 61 in [5] (66 in Additional file 13), the citrate exchange reaction, was considered reversible. This resulted in a network with in total 68 fluxes, 62 intracellular metabolites and 10 extracellular metabolites (glucose, glycerol, ammonia, sulphate, citrate, 1,3-propanediol, acetate, carbon dioxide, oxygen and biomass). The number of free fluxes is 6, and these were chosen to be (numbering according to Additional file 13) flux 62 (glycerol exchange), 63 (glucose uptake), 65 (ammonia uptake), 66 (citrate exchange), 67 (acetate uptake) and 68 (oxygen uptake). Furthermore, there are 44 irreversible fluxes.

**Figure 13:**
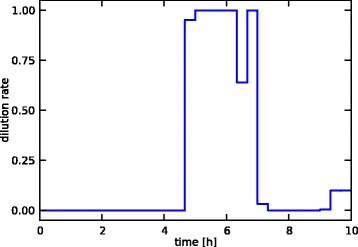
**Medium-scale case study input profile.** The dilution rate input profile for the continuous controlled bioreactor model of the medium-scale case study.

Measurements were generated by considering a continuous reactor set-up in which, after starting the reactor with a full medium, only glucose is added from a feed with a fixed concentration. The model equation (Equation (9)) is changed accordingly:

(53) $$ \begin{array}{lcrr}\frac{d\mathbf{x}(t)}{dt}& =& {\mathbf{S}}_{\mathrm{e}}\cdotp \mathbf{K}\cdotp \mathbf{u}(t)\cdotp {\mathbf{q}}_{\mathrm{bio}}^T\cdotp \mathbf{x}(t)+D\cdotp \left({\mathbf{x}}_{\mathrm{in}}-\mathbf{x}\right)& \end{array} $$

with *D* the dilution rate [1/h], which is controlled between 0 and 1 following the input profile in Figure 13, and **x**_in_ the (10×1) vector of feed concentrations [mmol/L] of the different metabolites. As only glucose is in the feed, with a concentration of 20, this vector is the following:

(54) $$ {\mathbf{x}}_{\mathrm{in}}=\left[\begin{array}{l}20.0\\ {}0.0\\ {}\vdots \\ {}0.0\end{array}\right] $$

The reference fluxes for the six free fluxes were the following:

(55) $$ \begin{array}{lcrr}{u}_{62,\mathrm{r}\mathrm{e}\mathrm{f}}& =& 0.0995& \end{array} $$

(56) $$ \begin{array}{lcrr}{u}_{63,\mathrm{r}\mathrm{e}\mathrm{f}}& =& 0.5605\cdotp \frac{c_{\mathrm{Gluc}}}{9.89+{c}_{\mathrm{Gluc}}}& \end{array} $$

(57) $$ \begin{array}{lcrr}{u}_{65,\mathrm{r}\mathrm{e}\mathrm{f}}& =& 0.122\cdotp \frac{c_{\mathrm{NH}3}}{0.1919+{c}_{\mathrm{NH}3}}& \end{array} $$

(58) $$ \begin{array}{lcrr}{u}_{66,\mathrm{r}\mathrm{e}\mathrm{f}}& =& 0.0207\cdotp \frac{c_{\mathrm{Cit}}}{5.575+{c}_{\mathrm{Cit}}}& \end{array} $$

(59) $$ \begin{array}{lcrr}{u}_{67,\mathrm{r}\mathrm{e}\mathrm{f}}& =& 0.8834\cdotp \frac{1}{10.0+{c}_{\mathrm{Ac}}}& \end{array} $$

(60) $$ \begin{array}{lcrr}{u}_{68,\mathrm{r}\mathrm{e}\mathrm{f}}& =& 0.438\cdotp \frac{c_{\mathrm{O}2}}{9.706+{c}_{\mathrm{O}2}}& \end{array} $$

The starting values for the concentrations

(61) $$ \begin{array}{c}\left[{c}_{\mathrm{Gluc}},{c}_{\mathrm{Glyc}},{c}_{\mathrm{NH}3},{c}_{\mathrm{SO}4},{c}_{\mathrm{Cit}},{c}_{\mathrm{PDO}},{c}_{\mathrm{Ac}},{c}_{\mathrm{CO}2},{c}_{\mathrm{O}2},\right.\\ {}\kern1em {\left.{c}_{\mathrm{BIOMASS}}\right]}^T\end{array} $$

in the simulation were chosen to be

(62) $$ \begin{array}{l}{\left[100,53.15,38.45,62.61,0,0,0,6.78,100,2.14\right]}^T\end{array} $$

**Figure 14:**
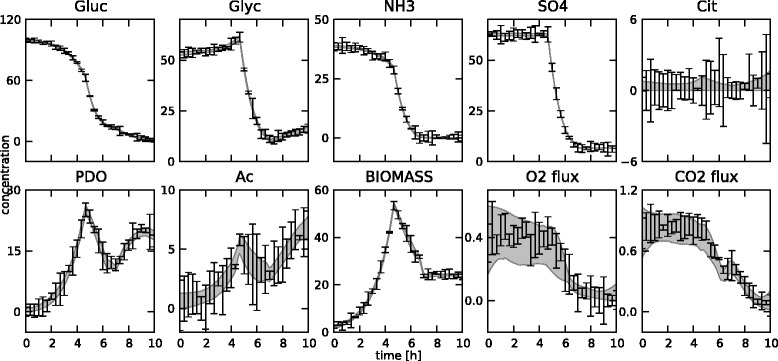
**Estimated state trajectories.** The estimated state trajectories for the medium-scale network with fixed **K** matrix, along with the simulated data on which the estimation is based.

**Figure 15:**
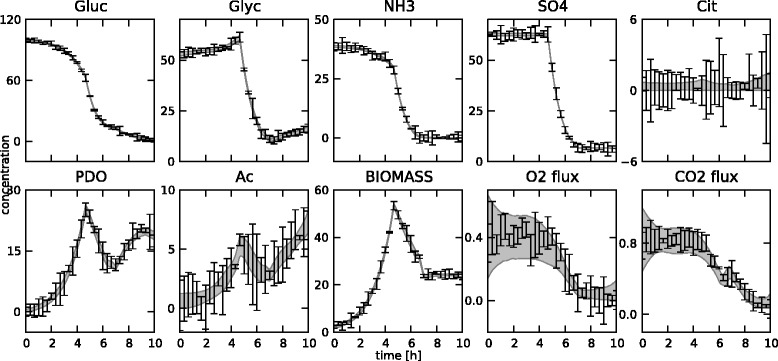
**Estimated state trajectories.** The estimated state trajectories for the medium-scale network with optimized **K** matrix, along with the simulated data on which the estimation is based.

**Figure 16:**
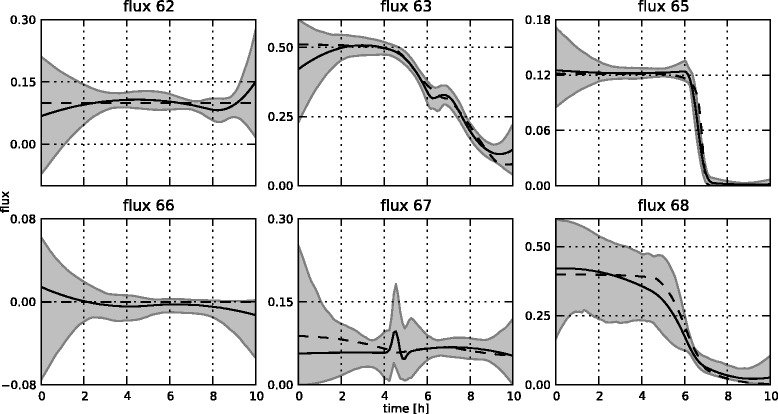
**Estimated flux profiles.** The estimated free flux profiles for the medium-scale network with fixed **K** matrix (in full line), including 95% confidence regions. For brevity, only the free fluxes are shown. All other fluxes can be calculated through the **K** matrix. In dashes are the reference profiles which were used to simulate the measurements.

**Figure 17:**
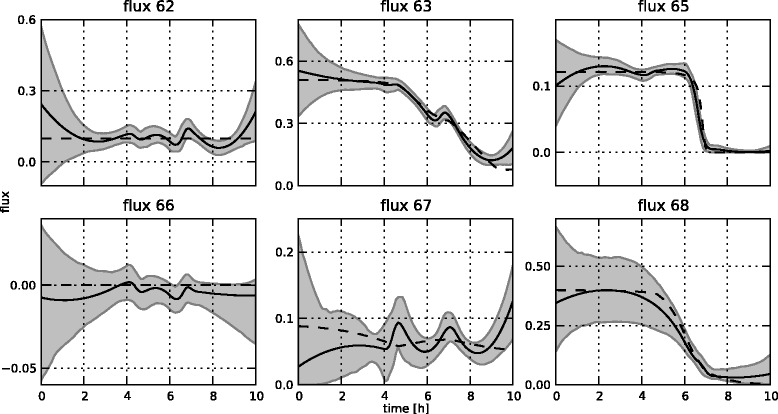
**Estimated flux profiles.** The estimated free flux profiles for the medium-scale network with optimized **K** matrix (in full line), including 95% confidence regions. For brevity, only the free fluxes are shown. In dashes are the reference profiles which were used to simulate the measurements.

In total, 31 measurements at equidistant points between 0 and 10 hours were generated for 8 concentrations (glucose, glycerol, ammonia, sulphate, citrate, 1,3-propanediol, acetate and biomass) and 2 fluxes (oxygen and carbon dioxide). In typical settings, oxygen and carbon dioxide concentrations are not measured directly, but their uptake and production fluxes, respectively, are measured through off-gas analysis on the bioreactor. The resulting set of 310 measurements is not shown separately, but can be found in Figures 14 and 15, along with the fitted states. The reference fluxes are shown in Figures 16 and 17, along with the estimated fluxes.

All reactions, all stoichiometric matrices, the irreversibility matrix, the null space basis matrix, and the simulated measurements and measurement variances for this case study are given in the Additional files 13, 14, 15, 16, 17, 18, 19, 20, 21 and 22.

### Results for the medium-scale network

The estimation of the fluxes in the medium-scale network was carried out with both a fixed **K** matrix, again the same one as used to simulate the measurements, and an optimized **K** matrix. The estimated fluxes for the fixed **K** are shown in Figure 16, the results for the optimal **K** matrix are shown in Figure 17. Also, the fitted measurements for both cases are presented in Figures 14 and 15. It is clear that the proposed methodology is also able to estimate the fluxes in a larger network successfully. The confidence bounds are in this case, however, much wider than in the small-scale case. This is due to the larger amount of measurement noise added to the simulated measurements. Due to the minimization of the *AIC* criterion, this is the best possible fit while at the same time keeping the uncertainty as low as possible. Also, the difference between the estimates with a fixed and an optimal **K** matrix is again very small. This again confirms the fact that the algorithm can be successfully used in the optimal **K** mode, i.e., without making an a priori choice on the free fluxes, as the results are very similar in the two cases. This is an important advantage of this method as in practice the set of free fluxes is not known.

### Computational complexity of the algorithm

The time needed to solve the dMFA problem using this methodology is the product of on the one hand the total number of optimization problems to solve, and on the other hand the average time per optimization problem. If only one knot addition is allowed for every measurement time point which is added, and when making abstraction of the possibility that in the first iteration(s) no knot can be inserted, the total number of optimization problems equals *n*_time_·*d*, and thus scales linearly with both the number of measurement time points and the number of free fluxes. The average time per optimization problem is harder to assess, as this is dependent on the total number of variables, as indicated in Table 4, but also on the number of non-zeros in the Jacobians and Hessians, and other characteristics of the NLP solver used. It is important to notice that the total number of fluxes does not influence the computational complexity, so this methodology should be usable also for larger networks with more fluxes, but a number of free fluxes which is still considerable. Typical networks used for metabolic flux analysis have a number of fluxes in the range of 100, but a number of free fluxes that is typically in the region of 10.

**Table 4:** **CPU times for the two case studies, both with fixed K and optimized K**

**Case study**	**K matrix**	**Running time in seconds**
Small-scale	Fixed	31.2
Small-scale	Optimal	51.0
Medium-scale	Fixed	494.0
Medium-scale	Optimal	1771.4

**Figure 18:**
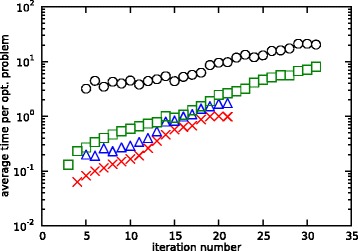
**Average CPU time per optimization problem.** The average CPU times per optimization problem plotted as a function of the iteration number. In red crosses for the small-scale network with fixed **K**, in blue triangles for the small-scale network with optimal **K**, in green squares for the medium-scale network with fixed **K**, and in black circles for the medium-scale network with optimal **K**.

To give a general idea about the computational complexity of the algorithm, the total CPU times of the estimations for the small-scale and medium-scale network for both fixed and optimal **K** matrices are shown in Table 4. These times are attained when running the algorithm on one core of an eight-core Intel i7-3770 CPU at 3.40 Ghz. Also, the average time per optimization problem over the different iterations is plotted as a function of the iteration number for the four cases, i.e., small- and medium-scale with fixed and free **K**, in Figure 18. Based on these findings, a general guideline to keep CPU times reasonable is to keep the time horizon under consideration small, i.e., with not too many measurement time points, as this will reduce the total number of optimization problems. Furthermore, every added time point increases the computational cost per optimization problem exponentially, so it is better to only use data around a specific region of interest, instead of using all possible data in a very wide time horizon. One could, for example, run the algorithm on a coarse subset of the measurements and choose, based on the results, a specific region of interest where a finer grid of measurements is used to get a better estimate. Although it is not investigated in this work, the algorithm also exhibits very nice possibilities for parallellization, as in every iteration of the algorithm *d* mutually independent optimization problems have to be solved. These can be easily assigned to different cores on a multi-core CPU. Finally, the automatic differentiation of the objective and constraint functions of the different subproblems can also be further exploited, as at this point, these are basically repeated when going from one iteration to the next. The only part which is added, though, is the part for the interval between the last and the newly added time point, while everything before this point stays the same. This initialization of the different subproblems offers opportunities for further reductions in CPU times in future research. Nevertheless, the algorithm can already at present perfectly run in real-time on standard equipment as bioprocesses typically involve multiple days.

## Conclusions

In this contribution, a novel systematic methodology for dynamic metabolic flux analysis, based on B-spline parameterizations, has been presented. Because of the high degree of non-linearity in the estimation of the knot locations, an incremental knot insertion algorithm is proposed. By using this algorithm, at least an excellent local minimum is found. Furthermore, the algorithm is fully contained, as the user does not have to make any choices regarding the null space basis of the intracellular stoichiometric matrix. This methodology tackles the disadvantages of previous methods for dMFA by making sure that the estimates are smooth, that specific fluxes are estimated and that extra constraints can be taken into account. The algorithm is validated on a small-scale simulated case study in both a low noise and a realistic noise setting. In both cases, an accurate dynamic estimation of the fluxes is obtained. The algorithm was also tested on a more realistic network with 68 fluxes and 6 free fluxes. Although CPU times are longer, mainly due to a larger number of measurements, the algorithm was also able to successfully estimate the fluxes in this larger case study. The algorithm can still be run in real-time as biological processes are typically slow. To keep CPU times under control, the total number of measurements should be reduced if possible, as the CPU time per iteration tends to grow exponentially over the iterations. This can be done, e.g., by only considering time horizons which are of specific interest to the researcher. Possible further improvements of the algorithm, mainly in the regions of parallellization and subproblem initialization, are indicated.

## Additional files

Additional file 1
**List of reactions for the small-scale network.** Lists of reactions, intracellular and extracellular metabolites for the small-scale network, in Microsoft Office Word format.

Additional file 2
**Intracellular stoichiometric matrix for the small-scale network.** (4×7) **S**_int_ matrix for the small-scale network, in Microsoft Office Excel format.

Additional file 3
**Combined extracellular and biomass stoichiometric matrix for the small-scale network.** (4×7) **S**_e_ matrix for the small-scale network, in Microsoft Office Excel format.

Additional file 4
**Irreversibility matrix for the small-scale network.** (5×7) **I**_irr_ matrix for the small-scale network, in Microsoft Office Excel format.

Additional file 5
**Null space basis for the** ***145*** **case of the small-scale case study.** (7×3) **K** matrix for the *145* case of the small-scale case study, in Microsoft Office Excel format.

Additional file 6
**Null space basis for the** ***367*** **case of the small-scale case study.** (7×3) **K** matrix for the *367* case of the small-scale case study, in Microsoft Office Excel format.

Additional file 7
**Null space basis for the** ***orthonormal*** **case of the small-scale case study.** (7×3) **K** matrix for the *orthonormal* case of the small-scale case study, in Microsoft Office Excel format.

Additional file 8
**Time points where measurements are taken in the small-scale case study.** (21×1) vector of time points where measurements are taken in the small-scale case study, in Microsoft Office Excel format.

Additional file 9
**Measurements for the low noise setting in the small-scale case study.** (21×4) matrix representing the measurements of the different concentrations (as indicated in Additional file 1) along the columns at the different measurement time points along the rows, for the low noise setting in the small-scale case study, in Microsoft Office Excel format.

Additional file 10
**Measurement variances for the low noise setting in the small-scale case study.** (21×4) matrix representing the variances of the measurements of the different concentrations (as indicated in Additional file 1) along the columns at the different measurement time points along the rows, for the low noise setting in the small-scale case study, in Microsoft Office Excel format.

Additional file 11
**Measurements for the realistic noise setting in the small-scale case study.** (21×4) matrix representing the measurements of the different concentrations (*A*_*ext*_, *E*_*ext*_, *F*_*ext*_ and Biomass) along the columns at the different measurement time points along the rows, for the realistic noise setting in the small-scale case study, in Microsoft Office Excel format.

Additional file 12
**Measurement variances for the realistic noise setting in the small-scale case study.** (21×4) matrix representing the variances of the measurements of the different concentrations (*A*_*ext*_, *E*_*ext*_, *F*_*ext*_ and Biomass) along the columns at the different measurement time points along the rows, for the realistic noise setting in the small-scale case study, in Microsoft Office Excel format.

Additional file 13
**List of reactions for the medium-scale network.** Lists of reactions, intracellular and extracellular metabolites for the medium-scale network, in Microsoft Office Word format.

Additional file 14
**Intracellular stoichiometric matrix for the medium-scale network.** (62×68) **S**_int_ matrix for the medium-scale network, in Microsoft Office Excel format.

Additional file 15
**Combined extracellular and biomass stoichiometric matrix for the medium-scale network.** (10×68) **S**_e_ matrix for the medium-scale network, in Microsoft Office Excel format.

Additional file 16
**Irreversibility matrix for the medium-scale network.** (44×68) **I**_irr_ matrix for the medium-scale network, in Microsoft Office Excel format.

Additional file 17
**Null space basis for medium-scale case study.** (68×6) **K** matrix for the medium-scale case study, in Microsoft Office Excel format.

Additional file 18
**Time points where measurements are taken in the medium-scale case study.** (31×1) vector of time points where measurements are taken in the medium-scale case study, in Microsoft Office Excel format.

Additional file 19
**Concentration measurements for the medium-scale case study.** (31×8) matrix representing the measurements of the different concentrations (glucose, glycerol, ammonia, sulphate, citrate, 1,3-propanediol, acetate and biomass) along the columns at the different measurement time points along the rows, for the medium-scale case study, in Microsoft Office Excel format.

Additional file 20
**Concentration measurement variances for the medium-scale case study.** (31×8) matrix representing the variances of the measurements of the different concentrations (glucose, glycerol, ammonia, sulphate, citrate, 1,3-propanediol, acetate and biomass) along the columns at the different measurement time points along the rows, for the medium-scale case study, in Microsoft Office Excel format.

Additional file 21
**Flux measurements for the medium-scale case study.** (31×2) matrix representing the measurements of the oxygen and carbon dioxide fluxes along the columns at the different measurement time points along the rows, for the medium-scale case study, in Microsoft Office Excel format.

Additional file 22
**Flux measurement variances for the medium-scale case study.** (31×2) matrix representing the variances of the measurements of oxygen and carbon dioxide fluxes along the columns at the different measurement time points along the rows, for the medium-scale case study, in Microsoft Office Excel format.
