# Supplementary material for: Dynamic estimation of specific fluxes in metabolic networks using non-linear dynamic optimization
Source: BMC Syst Biol. 2014 Dec 3;8:132. doi: 10.1186/s12918-014-0132-0 (PMC4280005; doi:10.1186/s12918-014-0132-0)
Supplement: Additional file 1 — List of reactions for the small-scale network. Lists of reactions, intracellular and extracellular metabolites for the small-scale network, in Microsoft Office Word format. [file 12918_2014_132_MOESM1_ESM.docx]

# List of reactions for the small-scale network:

**1**: 1 A_ext_ -> 1 A

**2**: 1 A -> 1 B

**3**: 1 A <-> 1 C

**4**: 1 B + 1 E_ext_ -> 2 D

**5**: 2 B -> 1 C + 1 F_ext_

**6**: 1 C <-> 1 D

**7**: 1 D -> 1 BIO

# List of intracellular metabolites, same ordering as the rows of the intracellular stoichiometric matrix:

A

B

C

D

# List of extracellular metabolites, same ordering as the rows of the combined extracellular and biomass stoichiometric matrix:

A_ext_

E_ext_

F_ext_

BIO
