# Supplementary material for: Dynamic estimation of specific fluxes in metabolic networks using non-linear dynamic optimization
Source: BMC Syst Biol. 2014 Dec 3;8:132. doi: 10.1186/s12918-014-0132-0 (PMC4280005; doi:10.1186/s12918-014-0132-0)
Supplement: Additional file 13 — List of reactions for the medium-scale network. Lists of reactions, intracellular and extracellular metabolites for the medium-scale network, in Microsoft Office Word format. [file 12918_2014_132_MOESM13_ESM.docx]

# List of reactions for the medium-scale network, based on Leighty (2011):

**1**: 0.488 Ala + 0.281 Arg + 0.229 Asn + 0.229 Asp + 0.087 Cys + 0.250 Glu + 0.250 Gln + 0.582 Gly + 0.090 His + 0.276 Ile + 0.428 Leu + 0.326 Lys + 0.146 Met + 0.176 Phe + 0.210 Pro + 0.205 Ser + 0.241 Thr + 0.054 Trp + 0.131 Tyr + 0.402 Val + 0.205 G6P + 0.071 F6P + 0.754 R5P + 0.129 GAP + 0.619 3PG + 0.051 PEP + 0.083 Pyr + 2.510 AcCoA + 0.087 AKG + 0.340 OAC + 0.443 MEETHF + 33.247 ATP + 5.363 NADPH -> 39.68 BIOMASS + 1.455 NADH

**2**: 1 G6P <-> 1 F6P

**3**: 1 F6P + 1 ATP -> 1 FBP

**4**: 1 FBP <-> 1 DHAP + 1 GAP

**5**: 1 DHAP <-> 1 GAP

**6**: 1 GAP <-> 1 3PG + 1 ATP + 1 NADH

**7**: 1 3PG <-> 1 PEP

**8**: 1 PEP -> 1 Pyr + 1 ATP

**9**: 1 G6P -> 1 6PG + 1 NADPH

**10**: 1 6PG -> 1 Ru5P + 1 CO2 + 1 NADPH

**11**: 1 Ru5P <-> 1 X5P

**12**: 1 Ru5P <-> 1 R5P

**13**: 1 X5P <-> 1 TK-C2 + 1 GAP

**14**: 1 F6P <-> 1 TK-C2 + 1 E4P

**15**: 1 S7P <-> 1 TK-C2 + 1 R5P

**16**: 1 F6P <-> 1 TA-C3 + 1 GAP

**17**: 1 S7P <-> 1 TA-C3 + 1 E4P

**18**: 1 Pyr -> 1 AcCoA + 1 CO2 + 1 NADH

**19**: 1 OAC + 1 AcCoA -> 1 Cit

**20**: 1 Cit <-> 1 ICit

**21**: 1 ICit <-> 1 AKG + 1 CO2 + 1 NADPH

**22**: 1 AKG -> 1 SucCoA + 1 CO2 + 1 NADH

**23**: 1 SucCoA <-> 1 Suc + 1 ATP

**24**: 1 Suc <-> 1 Fum + 1 FADH2

**25**: 1 Fum <-> 1 Mal

**26**: 1 Mal <-> 1 OAC + 1 NADH

**27**: 1 PEP + 1 CO2 -> 1 OAC

**28**: 1 AcCoA <-> 1 Ac + 1 ATP

**29**: 1 DHAP + 1 NADH <-> 1 Glyc3P

**30**: 1 Glyc3P -> 1 Glyc

**31**: 1 Glyc -> 1 HPA

**32**: 1 HPA + 1 NADPH -> 1 PDO

**33**: 1 AKG + 1 NADPH + 1 NH3 -> 1 Glu

**34**: 1 Glu + 1 ATP + 1 NH3 -> 1 Gln

**35**: 1 Glu + 1 ATP + 2 NADPH -> 1 Pro

**36**: 1 Glu + 1 CO2 + 1 Gln + 1 Asp + 1 AcCoA + 5 ATP + 1 NADPH -> 1 Arg + 1 AKG + 1 Fum + 1 Ac

**37**: 1 OAC + 1 Glu -> 1 Asp + 1 AKG

**38**: 1 Asp + 2 ATP + 1 NH3 -> 1 Asn

**39**: 1 Pyr + 1 Glu -> 1 Ala + 1 AKG

**40**: 1 3PG + 1 Glu -> 1 Ser + 1 AKG + 1 NADH

**41**: 1 Ser <-> 1 Gly + 1 MEETHF

**42**: 1 Thr <-> 1 Gly + 1 AcCoA + 1 NADH

**43**: 1 Ser + 1 AcCoA + 3 ATP + 4 NADPH + 1 SO4 -> 1 Cys + 1 Ac

**44**: 1 Asp + 1 Pyr + 1 Glu + 1 SucCoA + 1 ATP + 2 NADPH -> 1 LL-DAP + 1 AKG + 1 Suc

**45**: 1 LL-DAP -> 1 Lys + 1 CO2

**46**: 1 Asp + 2 ATP + 2 NADPH -> 1 Thr

**47**: 1 Asp + 1 METHF + 1 Cys + 1 SucCoA + 1 ATP + 2 NADPH -> 1 Met + 1 Pyr + 1 Suc + 1 NH3

**48**: 1 Pyr + 1 Pyr + 1 Glu + 1 NADPH -> 1 Val + 1 CO2 + 1 AKG

**49**: 1 AcCoA + 1 Pyr + 1 Pyr + 1 Glu + 1 NADPH -> 1 Leu + 1 CO2 + 1 CO2 + 1 AKG + 1 NADH

**50**: 1 Thr + 1 Pyr + 1 Glu + 1 NADPH -> 1 Ile + 1 CO2 + 1 AKG + 1 NH3

**51**: 2 PEP + 1 E4P + 1 Glu + 1 ATP + 1 NADPH -> 1 Phe + 1 CO2 + 1 AKG

**52**: 2 PEP + 1 E4P + 1 Glu + 1 ATP + 1 NADPH -> 1 Tyr + 1 CO2 + 1 AKG + 1 NADH

**53**: 1 Ser + 1 R5P + 2 PEP + 1 E4P + 1 Gln + 3 ATP + 1 NADPH -> 1 Trp + 1 CO2 + 1 GAP + 1 Pyr + 1 Glu

**54**: 1 R5P + 1 FTHF + 1 Gln + 1 Asp + 5 ATP -> 1 His + 1 AKG + 1 Fum + 2 NADH

**55**: 1 MEETHF + 1 NADH -> 1 METHF

**56**: 1 MEETHF -> 1 FTHF + 1 NADPH

**57**: 1 NADH + 0.5 O2 -> 3 ATP

**58**: 1 FADH2 + 0.5 O2 -> 2 ATP

**59**: 1 ATP ->

**60**: 1 CO2 -> 1 CO2[e]

**61**: 1 PDO -> 1 PDO[e]

**62**: 1 Glyc <-> 1 Glyc[e]

**63**: 1 Gluc[e] + 1 ATP -> 1 G6P

**64**: 1 SO4[e] -> 1 SO4

**65**: 1 NH3[e] -> 1 NH3

**66**: 1 Cit[e] <-> 1 Cit

**67**: 1 Ac -> 1 Ac[e]

**68**: 1 O2[e] -> 1 O2

# List of intracellular metabolites, same ordering as the rows of the intracellular stoichiometric matrix:

G6P

F6P

ATP

FBP

DHAP

GAP

3PG

NADH

PEP

Pyr

6PG

NADPH

Ru5P

CO2

X5P

R5P

TK-C2

E4P

S7P

TA-C3

AcCoA

OAC

Cit

ICit

AKG

SucCoA

Suc

Fum

FADH2

Mal

Ac

Glyc3P

Glyc

HPA

PDO

NH3

Glu

Gln

Pro

Asp

Arg

Asn

Ala

Ser

Gly

MEETHF

Thr

SO4

Cys

LL-DAP

Lys

METHF

Met

Val

Leu

Ile

Phe

Tyr

Trp

FTHF

His

O2

# List of extracellular metabolites, same ordering as the rows of the combined extracellular and biomass stoichiometric matrix:

Gluc[e]

Glyc[e]

NH3[e]

SO4[e]

Cit[e]

PDO[e]

Ac[e]

CO2[e]

O2[e]

BIOMASS
